# LRFN5 and OLFM4 as novel potential biomarkers for major depressive disorder: a pilot study

**DOI:** 10.1038/s41398-023-02490-7

**Published:** 2023-06-06

**Authors:** Ke Xu, Peng Zheng, Shuang Zhao, Jiubing Wang, Jinzhou Feng, Yi Ren, Qi Zhong, Hanping Zhang, Xiangyu Chen, Jianjun Chen, Peng Xie

**Affiliations:** 1 Department of Neurology, The First Affiliated Hospital of Chongqing Medical University, Chongqing, China; 2 National Health Commission Key Laboratory of Diagnosis and Treatment on Brain Functional Diseases, The First Affiliated Hospital of Chongqing Medical University, Chongqing, China; 3 Department of Pathophysiology, Chongqing Medical University, Chongqing, China; 4 Department of Clinical Laboratory, Chongqing Mental Health Centre, Chongqing, China; 5 Institute of Life Sciences, Chongqing Medical University, Chongqing, China

**Keywords:** Depression, Diagnostic markers

## Abstract

Evidences have shown that both LRFN5 and OLFM4 can regulate neural development and synaptic function. Recent genome-wide association studies on major depressive disorder (MDD) have implicated LRFN5 and OLFM4, but their expressions and roles in MDD are still completely unclear. Here, we examined serum concentrations of LRFN5 and OLFM4 in 99 drug-naive MDD patients, 90 drug-treatment MDD patients, and 81 healthy controls (HCs) using ELISA methods. The results showed that both LRFN5 and OLFM4 levels were considerably higher in MDD patients compared to HCs, and were significantly lower in drug-treatment MDD patients than in drug-naive MDD patients. However, there were no significant differences between MDD patients who received a single antidepressant and a combination of antidepressants. Pearson correlation analysis showed that they were associated with the clinical data, including Hamilton Depression Scale score, age, duration of illness, fasting blood glucose, serum lipids, and hepatic, renal, or thyroid function. Moreover, these two molecules both yielded fairly excellent diagnostic performance in diagnosing MDD. In addition, a combination of LRFN5 and OLFM4 demonstrated a better diagnostic effectiveness, with an area under curve of 0.974 in the training set and 0.975 in the testing set. Taken together, our data suggest that LRFN5 and OLFM4 may be implicated in the pathophysiology of MDD and the combination of LRFN5 and OLFM4 may offer a diagnostic biomarker panel for MDD.

## Introduction

Major depressive disorder (MDD) is the most prevalent psychiatric disorder with high mortality and disability, and is one of the main causes of suicide [1, 2]. By 2030, depression is predicted to be one of the leading causes of illness burden globally [3]. However, the etiology of MDD is still not fully understood, and the effects of current treatments used in clinical practice are not satisfactory [4–6]. Moreover, antidepressant treatments are often conducted behind the onset of MDD and are afflicted with side effects. An approach to circumvent these limitations is to explore novel clues of the pathogenesis of MDD and identify valuable biomarkers to support an objective diagnosis of MDD.

Leucine-rich repeat and fibronectin type III domain containing 5 (LRFN5) and olfactomedin 4 (OLFM4) have been suggested as significant risk factors for MDD in recent studies on genome-wide association (GWAS) and copy number variation [7–9]. LRFN5 belongs to a family of five small transmembrane protein genes involved in the development, organization, and plasticity of synapses [10–12]. And LRFN5 has another synonym which stands for synaptic adhesion-like molecule 5 (SALM5). SALMs are newly characterized adhesion molecules predominantly expressed in the brain contributing to neurite outgrowth and synapse formation. The five members of the SALM family are type I transmembrane proteins with an extracellular part consisting of an Ig-like domain (leucine-rich) and a fibronectin type III domain [13]. According to previous research, molecules encoded by LRFN5 are similar to those that create adhesive synapses, can cause excitatory and inhibitory presynaptic differentiation in contacting axons, and regulate synaptic strength [14]. In addition, by attaching to the herpes virus entry mediator, LRFN5 restricts the T cell response and neuroinflammation. As for OLFM4, it is a member of a well-conserved olfactomedin domain-containing glycoprotein family [15]. Currently, five members of this family have been identified in humans: OLFM1, OLFM2, OLFM3, OLFM4, and MYOC [16, 17]. OLFM4 is also a neutrophil-specific granule protein (humans and mice) and plays an important role in innate immunity, and inflammation, as well as in neurodevelopment and synaptic function [18], such as latrophilins forming trans-cellular complexes with neurexins [19] and regulating the number of glutamatergic synapses [20]. These studies indicated that the disturbances of LRFN5 and OLFM4 might involve immune and neuronal functions. A recent study analyzed the shared genetic architecture of the frequently co-morbid disorders MDD, insomnia, and chronic pain, and found jointly associated loci including 13q14.3 of gene OLFM4 and 14q21.1 of gene LRFN5 [21]. However, till now, very little is known about the expression and function of LRFN5 and OLFM4 in patients with depression. Therefore, further studies on these two molecules in MDD are required.

Nowadays, the diagnosis of MDD is still reliant on symptom ratings that are mostly subjective and lack any molecular basis, which restricts the development of objective diagnostic tools [22, 23]. Peripheral tissues from patients, such as blood, are being used more often to study psychiatric illness, and certain molecular mediators identified in these studies may serve as possible disease biomarkers [24, 25]. Thus, serum studies are required to learn about the possible function of LRFN5 and OLFM4 in MDD and whether they hold promise as biomarkers for objectively diagnosing MDD.

In this study, we measured the serum levels of LRFN5 and OLFM4 in MDD patients and HCs. The purposes of our current study included: (i) to determine whether serum levels of LRFN5 and OLFM4 were altered in drug-naive MDD patients; (ii) to observe their putative changes during antidepressants treatment; (iii) to explore the correlations between LRFN5/OLFM4 and clinical parameters; (iv) to assess whether these two molecules could be used as potential biomarkers for MDD and treatment responsiveness.

## Materials and methods

### Study population

The study was approved by the local ethics committee of Chongqing Medical University. Before taking blood samples, each participant gave their written informed permission. Between November 2021 and June 2022, 270 subjects (189 MDD patients and 81 healthy controls (HCs)) were enrolled. According to the Diagnostic and Statistical Manual of Mental Disorders-version IV criteria and the International Statistical Classification of Diseases and Related Health Problems criteria, 10th revision, the diagnosis of MDD was confirmed by two qualified psychiatrists. All MDD inpatients were enrolled from the First Affiliated Hospital of Chongqing Medical University’s Department of Psychiatry. To further investigate the effects of antidepressants on the levels of these molecules, patients with MDD were split into drug-naive MDD (DN-MDD; *n* = 99) group and drug-treatment MDD (DT-MDD; *n* = 90) group. The DN-MDD subgroup was defined as first-episode MDD and never received any antidepressant therapy, and DT-MDD subgroup was defined as having only ever undergone antidepressant therapy. During the same period, the Medical Examination Center, First Affiliated Hospital of Chongqing Medical University was used to recruit HCs. Considering that peripheral expression of OLFM4 was abnormal in some other diseases [26, 27], this study excluded subjects with other physical conditions. And the HCs with no previous neurological, DSM-IV Axis I/II, or medical illness. HCs were age-matched and sex-matched with MDD patients.

### Clinical data collection

The 17-item Hamilton Depression Scale (HAMD) was used to assess the severity of depression in MDD patients based on their basic clinical documentation. Clinical data were extracted from the patient discharge letters, including fasting blood glucose, liver function (alanine aminotransferase (ALT), aspartate aminotransferase (AST), and total bilirubin), renal function (urea nitrogen, creatinine, and uric acid (UA)), serum lipids (total cholesterol (TC), low-density lipoprotein cholesterol (LDL-C), high-density lipoprotein cholesterol (HDL-C), and triglyceride (TG)), thyroid function (triiodothyronine (T3), thyroxine, free T3, and free thyroxine). These additional laboratory tests were immediately carried out after assessing the individual score of HAMD. Meanwhile, age, sex, duration of illness, marital status (single/married/divorced/widowed), levels of education (low/middle/high), drinking status (never/moderate/heavy), and smoking status (never/moderate/heavy) were also collected. Detailed information on antidepressant usage was collected in the DT-MDD subgroup. Moreover, considering that combination antidepressant pharmacotherapies are also frequently used to treat MDD [28], the DT-MDD group was further classified as single and combined (≥2) antidepressant groups.

### Blood sampling

Similar to our previous study [29], after assessing the HAMD score, venous blood samples were drawn into coagulant tubes, and serum was then isolated by centrifugation at 3000 × *g* for 15 min under room temperature, aliquoted, and stored at −80 °C until use.

### Serum LRFN5 and OLFM4 concentrations detection

Total LRFN5 and OLFM4 were assessed using commercially available high-sensitivity ELISA kits from MEIMIAN (Jiangsu, China) by two blind researchers. We utilized every ELISA kit as directed by the manufacturer’s instructions. The reference standard was used on each ELISA plate to make a standard curve. For LRFN5, eight standards of different concentrations from 800 pg/mL to 0 pg/mL (800, 400, 200, 150, 100, 50, 10, and 0 pg/mL) were prepared. And for OLFM4, six standards of different concentrations from 480 pg/mL to 0 pg/mL for OLFM4 (480, 240, 120, 60, 30, and 0 pg/mL) were prepared. Described as in the flow chart (Fig. S1), fifty microliters of diluted serum samples (1:4) or standard were dispensed into the wells. The plate was sealed and incubated for 30 min at 37 °C on a plate shaker set to 400 rpm. Thereafter, each well was incubated with 50 μL of Streptavidin-HRP on a shaker at 37 °C after being washed five times with 350 μL of wash buffer (1×) for 30 s. Following the last wash, 100 μL of 3, 3’, 5, 5’-tetramethylbenzidine (TMB) development solution was added to each well and incubated for 10 min at 37 °C in the dark. A total of 50 μL of stop solution was then added for 1 min at room temperature on a plate shaker set to 400 rpm, resulting in a blue to yellow color change. Absorbance was read at 450 nm using an ELISA reader (Bio-Rad, Hercules, CA, USA). The results were expressed in pg/mL according to the established standard curve. The LRFN5 assay’s detection limit was 20 pg/mL, whereas the OLFM4 assay’s detection limit was 12 pg/mL. In addition, sample collection and storage time did not differ among groups.

### Statistical analysis

The Student’s t-test, Chi-squared test, nonparametric Mann Whitney U test, or one-way analysis of variance (one-way ANOVA) was used when appropriate. In one-way ANOVA, Tamhane’s T2 or Bonferroni post hoc analysis was performed if a significant difference was found. Then, the linear support vector machine was used to evaluate the potential diagnostic effectiveness of LRFN5 and OLFM4, controlling for the effects of sex and age. LIBSVM toolbox with default parameter values was applied to conduct linear support vector machine classifier. The area under the receiver operating characteristic curve (AUC) was the evaluation index [30–32]: 1–0.9, 0.9–0.8, 0.8–0.7, 0.7–0.6, and 0.6–0.5 represented excellent, good, fair, poor, and fail classification performance, respectively. All analyses were conducted using SPSS 20.0, and a *p*-value less than 0.05 was regarded as statistically significant. Unless specified, results were expressed as mean ± standard deviation (SD).

## Results

### Sociodemographic and clinical information

Table S1 displayed the demographic information for all individuals, which included 81 HCs and 189 MDD patients (99 DN-MDD and 90 DT-MDD (response rate = 52.22%)). Between MDD patients and HCs, there was no significant difference in sex (*p* = 0.353) and age (*p* = 0.768). In comparison to HCs, MDD patients’ HAMD scores were considerably higher (*p* = 8.67E−39). The majority of patients were married, well-educated, and never drank or smoked (Table S2). In addition, the majority of the antidepressants prescribed to DT-MDD individuals were primarily selective serotonin reuptake inhibitors (SSRI), including escitalopram, sertraline, and fluoxetine.

### Serum levels of LRFN5 and OLFM4 between MDD and HCs groups

**Fig. 1:**
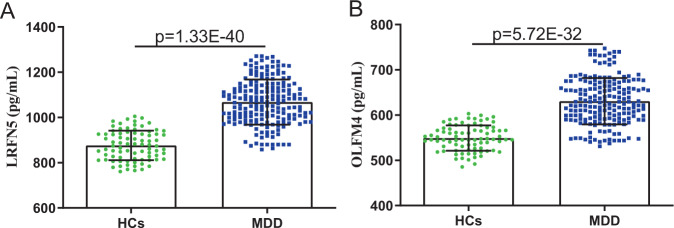
Concentrations of LRFN5 and OLFM4 in HCs and MDD groups. **A**, **B** Compared to HCs, MDD patients had significantly higher levels of LRFN5 (**A**) and OLFM4 (**B**). Data are presented as mean ± S.D. HCs healthy controls, MDD major depressive disorder.

As shown in Fig. 1, there was a significant difference in LRFN5 level between MDD patients (1068.29 ± 99.71) and HCs (876.43 ± 65.13) (*p* = 1.33E−40). The level of OLFM4 was also significantly increased in MDD patients (630.72 ± 51.15) than in HCs (549.29 ± 27.99) (*p* = 5.72E−32). Meanwhile, we compared the levels of fasting blood glucose, ALT, AST, total bilirubin, blood urea nitrogen, serum creatinine, UA, serum TC, LDL-C, HDL-C, TG, T3, thyroxine, free T3, and free thyroxine between HCs and MDD patients (Fig. S2). The results showed that the levels of the following biochemical indexes were significantly lower in MDD patients compared to HCs: fasting blood glucose (*p* = 0.007; Fig. S2A), TC (*p* = 8.61E−5; Fig. S2H), LDL-C (*p* = 1.98E−4; Fig. S2I), T3 (*p* = 0.009; Fig. S2L), thyroxine (*p* = 0.001; Fig. S2M), and free T3 (*p* = 0.030; Fig. S2N). The heat map consisting of these differential variables showed a consistent clustering pattern within the individual groups (Fig. S3).

### Effects of antidepressants and sex on LRFN5 and OLFM4 levels

**Fig. 2:**
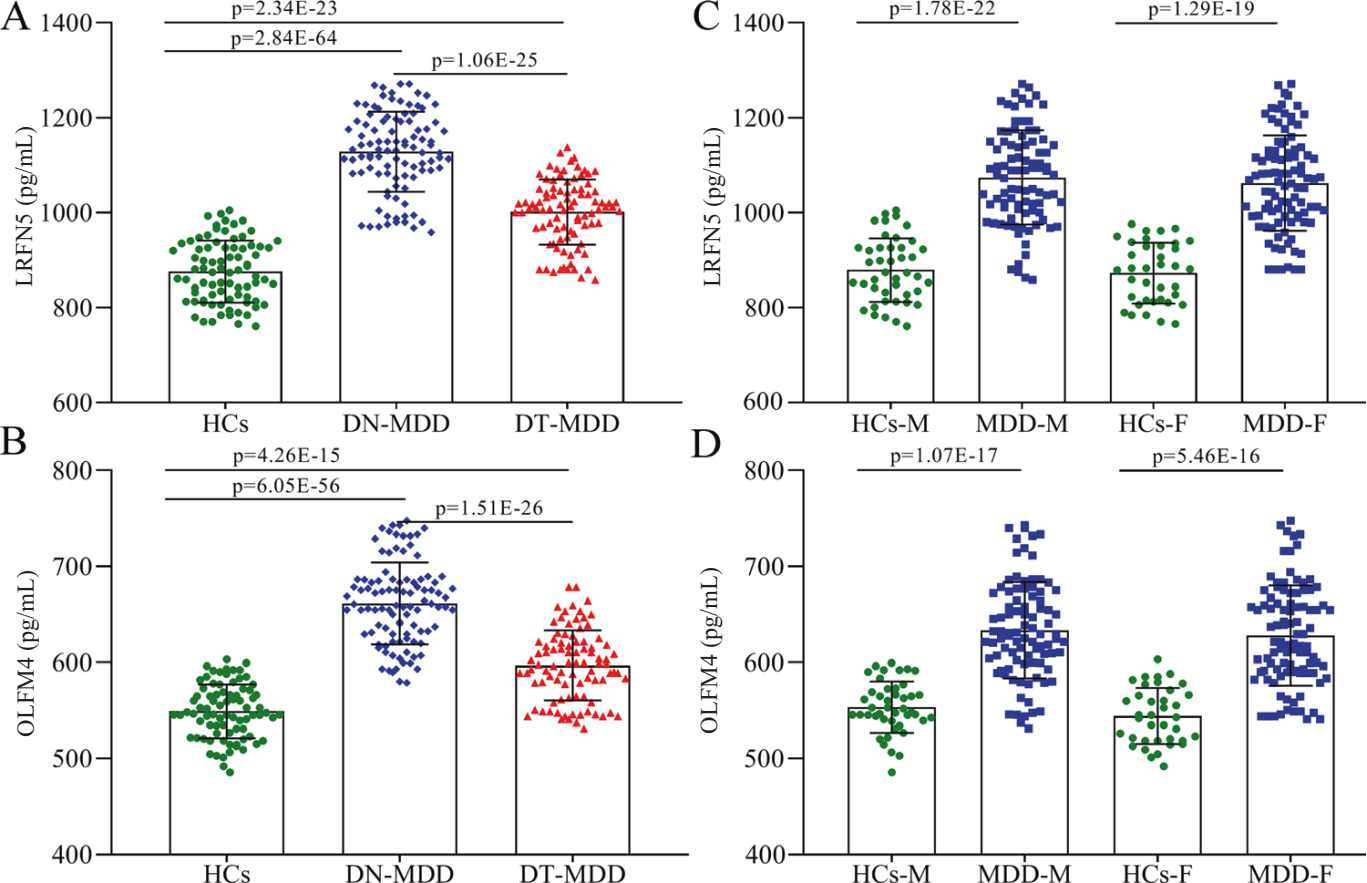
Effects of medication and sex on LRFN5 and OLFM4 levels. **A** effects of medication on LRFN5 level, antidepressants treatment could significantly decrease the level of LRFN5; **B** effects of medication on OLFM4 level, antidepressants treatment could significantly decrease the level of OLFM4; **C** sex had no significant impact on LRFN5 level; **D** sex had no significant impact on OLFM4 level. Data are presented as mean ± S.D. DN-MDD drug-naive major depressive disorder, DT-MDD drug-treatment major depressive disorder, HCs healthy controls, M male, F female.

At first, we examined the effects of antidepressants on LRFN5 and OLFM4 levels. As shown in Fig. 2A, both DN-MDD patients (*p* = 2.84E−64) and DT-MDD patients (*p* = 2.34E−23) showed significantly higher levels compared to HCs; the level of LRFN5 was significantly lower in DT-MDD patients than in DN-MDD patients (*p* = 1.06E−25). Meanwhile, we found that both DN-MDD patients (*p* = 6.05E−56) and DT-MDD patients (*p* = 4.26E−15) showed significantly higher levels of OLFM4 compared to HCs; the level of OLFM4 was significantly lower in DT-MDD patients than in DN-MDD patients (*p* = 1.51E−26) (Fig. 2B). These results indicated that antidepressants might have positive effects on the levels of LRFN5 and OLFM4.

Then, we examined the effects of sex on LRFN5 and OLFM4 levels. As shown in Fig. 2C, the levels of LRFN5 in male (*p* = 1.78E−22) and female (*p* = 1.29E−19) MDD patients were significantly different from that in their respective HCs (Fig. 2C). And the level of LRFN5 was similar between male and female MDD patients (*p* = 0.411), and also similar between male and female HCs (*p* = 0.663). Meanwhile, we found that the levels of OLFM4 in male (*p* = 1.07E−17) and female MDD (*p* = 5.46E−16) patients were significantly different from that in their respective HCs (Fig. 2D). And the level of OLFM4 was similar between male and female MDD patients (*p* = 0.456), and also similar between male and female HCs (*p* = 0.143). These results indicated that there were no sex differences on both LRFN5 and OLFM4 levels.

### Correlations between LRFN5/OLFM4 and other variables

To find out the potential correlations between these two differential molecules and other variables, Pearson correlation analysis was used here. As shown in Figure S4, the level of LRFN5 was significant negatively correlated with age (*r* = −0.388, *p* = 3.38E−08; Fig. S4A), duration of illness (*r* = −0.238, *p* = 0.001; Fig. S4C), fasting blood glucose (*r* = −0.173, *p* = 0.018; Fig. S4D), urea nitrogen (*r* = −0.149, *p* = 0.041; Fig. S4H), and TC (*r* = −0.162, *p* = 0.026; Fig. S4K) and positively correlated with HAMD score (*r* = 0.212, *p* = 0.0034; Fig. S4B).

**Fig. 3:**
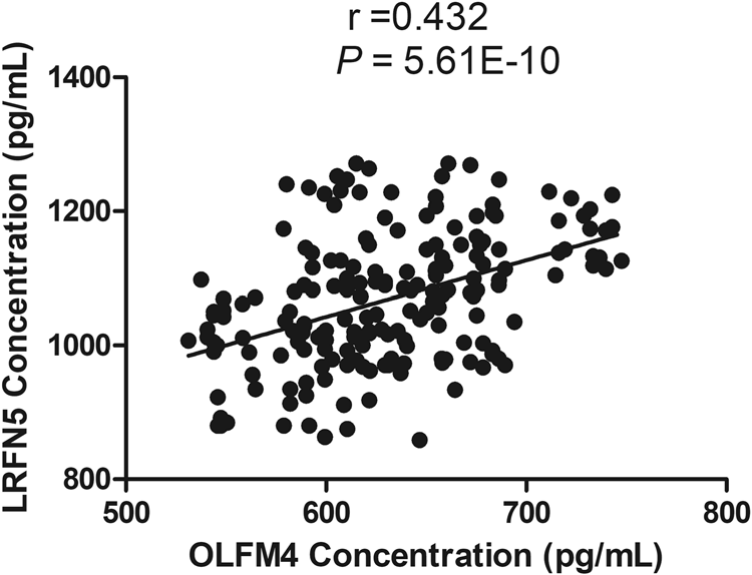
Correlation between LRFN5 and OLFM4. LRFN5 level was significantly positively correlated with OLFM4 level in serum.

**Fig. 4:**
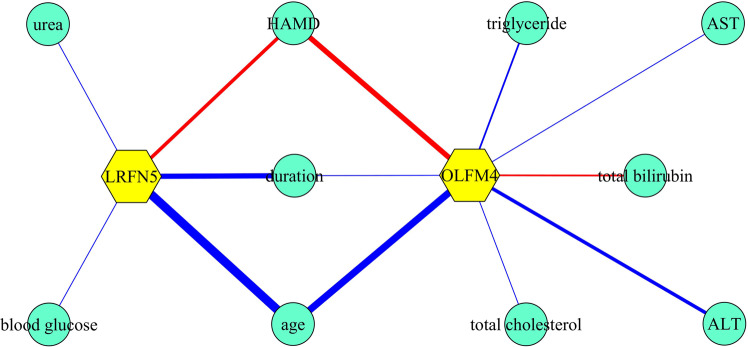
Correlations between LRFN5/OLFM4 and other variables. The blue and red lines represented negative and positive correlations, respectively. The thicker the line, the stronger the correlation between variables. ALT alanine aminotransferase, AST aspartate aminotransferase, *duration* duration of illness, HAMD Hamilton Depression Scale, *urea* urea nitrogen.

Moreover, as shown in Figure S5, the level of OLFM4 was significant negatively correlated with age (*r* = −0.776, *p* = 1.40E−55; Fig. S5A), duration of illness (*r* = −0.175, *p* = 0.016; Fig. S5C), ALT (*r* = −0.217, *p* = 0.003; Fig. S5F), AST (*r* = −0.182, *p* = 0.012; Fig. S5G), TC (*r* = −0.152, *p* = 0.037; Fig. S5K), and TG (*r* = −0.200, *p* = 0.006; Fig. S5L), and positively correlated with HAMD score (*r* = 0.213, *p* = 0.003; Fig. S5B) and total bilirubin (*r* = 0.147, *p* = 0.044; Fig. S5E). In addition, we found that there was a significant positive correlation between LRFN5 and OLFM4 (*r* = 0.432, *p* = 5.61E−10; Fig. 3). Moreover, the significant correlations between these variables were shown in Fig. 4.

### Effects of different treatment modalities on LRFN5 and OLFM4 levels

**Fig. 5:**
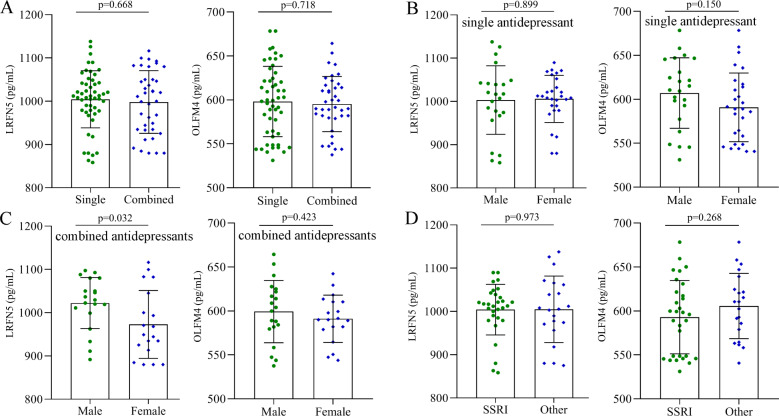
Effects of different treatment modalities on LRFN5 and OLFM4 levels. **A** effects of single antidepressant vs. combined antidepressants on LRFN5 and OLFM4 levels; **B** there was no sex-specific difference in LRFN5 and OLFM4 levels in patients receiving single antidepressant; **C** compared to male patients, female patients receiving combined antidepressants had a significantly lower level of LRFN5 and a similar level of OLFM4; **D** among the patients receiving single antidepressant, the levels of LRFN5 and OLFM4 were similar between patients receiving selective serotonin reuptake inhibitor (SSRI) and patients receiving other antidepressants. Data are presented as mean ± S.D.

The characteristics of antidepressants used in DT-MDD subjects are shown in Table S3, which included 51 receiving single antidepressants (30 SSRIs and 21 others) and 39 receiving combined antidepressants. The levels of LRFN5 (*p* = 0.668) and OLFM4 (*p* = 0.718) were similar between MDD patients receiving single antidepressants and MDD patients receiving combined antidepressants (Fig. 5A). Among the MDD patients receiving a single antidepressant, the levels of LRFN5 (*p* = 0.899) and OLFM4 (*p* = 0.150) were similar between male and female patients (Fig. 5B). Among the MDD patients receiving combined antidepressants, the significant difference on the level of LRFN5 (*p* = 0.032) was found between male and female patients, but not on the level of OLFM4 (*p* = 0.423, Fig. 5C). In addition, among the MDD patients receiving single antidepressant, patients receiving SSRI had the similar levels of LRFN5 (*p* = 0.973) and OLFM4 (*p* = 0.268) compared to patients receiving other antidepressants (Fig. 5D).

### LRFN5 and OLFM4 as potential biomarkers for diagnosing MDD

**Fig. 6:**
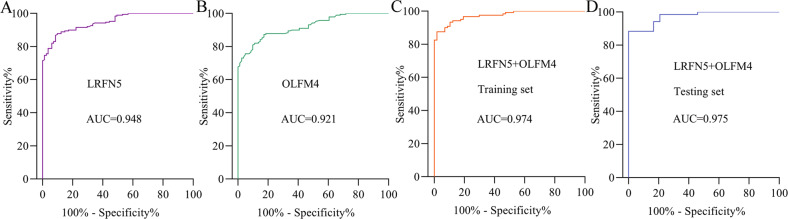
Diagnostic performances of LRFN5 and OLFM4 in diagnosing MDD. **A** area under the receiver operating characteristic curve (AUC) value of LRFN5 in diagnosing MDD patients; **B** AUC value of OLFM4 in diagnosing MDD patients; **C** AUC value of the combination of LRFN5 and OLFM4 in diagnosing MDD patients in training set; **D** AUC value of the combination of LRFN5 and OLFM4 in diagnosing MDD patients in testing set.

The diagnostic values of these two molecules were shown in Fig. 6. The AUC value of LRFN5 was 0.948 (sensitivity = 86.77%, specificity = 91.36%; Fig. 6A), and the AUC value of OLFM4 was 0.921 (sensitivity = 80.95%, specificity = 90.12%; Fig. 6B), suggesting the excellent diagnostic power in diagnosing MDD. To explore the diagnostic power of these two molecules when combined, the included subjects were randomly assigned to the training set and testing set. The training set was used to obtain a discriminative model, and the testing set was used to independently validate the diagnostic performance of the built model. The results showed that the combination of these two molecules could yield a better AUC value of 0.974 in training set (sensitivity = 87.72%, specificity = 94.17%; Fig. 6C). Moreover, the combination of these two molecules could also yield a better AUC value of 0.975 in testing set (sensitivity = 83.33%, specificity = 91.30%; Fig. 6D).

## Discussion

In the present study, we found significantly higher serum levels of LRFN5 and OLFM4 in MDD patients compared to HCs; and no sex-specific differences in both LRFN5 and OLFM4 levels were observed. Meanwhile, significantly lower levels of LRFN5 and OLFM4 in the DT-MDD group than in the DN-MDD group were found, which indicated that their levels might be partially reversed by antidepressant therapy. In addition, we further analyzed the effects of different treatment modalities on LRFN5 and OLFM4 levels; and the results showed that in MDD patients receiving combined antidepressants, the level of LRFN5 was significantly lower in female MDD patients than in male MDD patients. To the best of our knowledge, this is the first report showing the increased expression of LRFN5 and OLFM4 in the serum of MDD patients. Our results suggested that these two molecules might play an important role in the pathophysiology of depression.

Compared to a single biomarker, a biomarker panel consisting of multiple biomarkers could reduce the impact of variation between populations and subgroups, and then yield more accurate results. Previous studies found that the combination of multiple biomarkers yielded much better diagnostic effectiveness compared to every single biomarker [33, 34]. Our previous studies also reported similar results [35, 36]. In this study, we found that as single biomarkers, both LRFN5 and OLFM4 serum levels had great diagnostic accuracy for identifying MDD. Moreover, we found that the discriminative model consisting of these two molecules could yield a better AUC of 0.974 in the training set. And, this discriminative model could still yield an AUC of 0.975 in testing set, demonstrating the diagnostic robustness of this discriminative model. Therefore, our results suggested that the discriminative model consisting of LRFN5 and OLFM4 could be a “good” classifier of MDD patients and HCs.

LRFN5 encodes leucine-rich repeat (LRR) and fibronectin type 3 domain-containing protein 5. The LRFN family is primarily expressed in the central nervous system, which can cross the plasma membrane. It is believed that their extracellular domains take part in the cell-cell interactions required for both neuronal development [11, 37] and synapse formation [38]. The previous study showed that *Lrfn5* can induce both inhibitory and excitatory presynaptic differentiation in nearby neuronal cells [39], a process that might play a critical role in brain development and function [40]. Notably, presynaptic differentiation and synapse formation are associated with several neuropsychiatric disorders including MDD [41]. Moreover, the decreased expression of LRFN5 has been reported to promote neuroinflammation [13], and there were exact opposite expression patterns of LRFN5 in the brain versus the periphery [9]. A growing body of research also points to the role of neuroinflammation in the pathophysiology of MDD and resistance to antidepressant treatment [42]. Based on these findings, we postulated that LRFN5 might be decreased in the central nervous in contrast to its expression in serum and that neuroinflammation possibly serves as a link between LRFN5 and the onset of MDD.

OLFM4 is a member of the olfactomedin domain family. Multiple lines of evidence have suggested that it was involved in several biological processes, such as facilitating neurodevelopment, cell adhesion, intercellular connections, and protein–protein interactions [43]. An increasing body of research suggests that this family of proteins may be crucial to normal development and pathology [44]. For instance, OLFM1 plays a role in the biological process of brain ischemia and axon growth [45, 46]. In addition, it has been suggested that the disruption of neurodevelopment may be an etiology for depression [47, 48]. Our previous study has suggested that the abnormal metabolites in the psychological stress-induced depression model were related to neurodevelopment [49]. Furthermore, in this study, we found a significant correlation between the expression of OLFM4 and LRFN5 levels in serum. Neuroinflammation is an important inducer in the alteration of neurodevelopment, such as impacting synaptic plasticity and synaptogenesis [50]. To date, no reports have suggested that OLFM4 affects mood and behavior by regulating neuroinflammation and neurodevelopment, but it was reported to contribute to the severity of infectious disease [51]. Meanwhile, other pro-inflammatory markers like the high-mobility group box 1 (HMGB1) which is a highly conserved, ubiquitous protein present in the nuclei and cytoplasm of nearly all cell types, serve as a necessary and sufficient mediator of inflammation [52]. Elevated serum levels of HMGB1 were recently reported to be associated with depression after acute ischemic stroke [53]. HMGB1 was also found among Danger-/damage-associated molecular patterns which were reported for elevated serum levels in schizophrenia [54]. Therefore, future studies are needed to find out whether LRFN5 and OLFM4 are involved in the pathogenesis of MDD by regulating neuroinflammation.

Here, we found that there was no significant difference in the levels of LRFN5 and OLFM4 among MDD patients who used single or multiple antidepressants, and SSRIs or other antidepressants. The effects of antidepressants were not affected by sex in MDD patients who used single, SSRI, or other antidepressants, even though there was a significant difference in the level of LRFN5 between male and female MDD patients who used multiple antidepressants. These findings showed that neither a single nor a combination of drugs significantly affected the levels of LRFN5 and OLFM4.

In addition, we found that the clinical biochemical indices of fasting blood glucose, TC, LDL-C, T3, thyroxine, and free T3 were all considerably lower in MDD patients. Notably, it has been shown that both low and very high fasting blood glucose concentrations may be associated with depression [55, 56]. Meanwhile, the lower levels of lipids in MDD patients here were in line with some previous studies [57–59]. And abnormal thyroid function, especially hypothyroidism, has also been reported to relate to the severity and obvious psychopathological features of depression [60]. However, these results are contrary to several previous studies reporting that fasting blood glucose, TC, LDL-C, T3, thyroxine, and free T3 were higher in patients with MDD [61, 62]. This disparity might have resulted from differences in sample sizes, ethnicity, clinical characteristics of the MDD patients, and/or the detection methodology. Interestingly, we also found correlations between the levels of these aberrant clinical biochemical indices and the levels of LRFN5 or OLFM4, which further supported their involvement in depression.

Furthermore, polymorphic markers within or close to LRFN5 have also been reported to be associated with progressive autism and familial schizophrenia [63, 64]. In clinical practice, the initial symptoms of schizophrenia have some overlaps with the depressive features of MDD. Some signs of inflammation, such as IL-6 and TNF-α, overlap in both MDD and schizophrenia. And similar to our findings, these inflammatory markers in serum in drug-native patients with MDD seem to normalize following treatment with antidepressants [65, 66]. Meanwhile, schizophrenia may often be misdiagnosed as MDD and treated with inappropriate antidepressant therapy. Thus, it is necessary to further investigate the levels of these two molecules in other psychiatric disorders. The results will be helpful for further determining the utility of these biomarkers in differentiating MDD from other psychiatric disorders and identifying subgroups of patients with MDD.

As there is presently relatively little data concerning the relations of LRFN5 and OLFM4 to depression, the results of our study can provide a starting point for further investigating their relations. Given that the levels of LRFN5 and OLFM4 were elevated in the serum of patients with MDD, and lower in DT-MDD patients than in DN-MDD patients, they might be used as additional state or trait biomarkers for depression. Moreover, considering that there is mounting evidence showing that hippocampal atrophy is associated with MDD, already from the onset of MDD, and that antidepressants may block/reverse hippocampal atrophy [67, 68]. For example, antidepressant may upregulate BDNF which, in turn, increases neurogenesis within the hippocampus [69]. We speculate that antidepressants may also increase neurogenesis within the hippocampus via downregulating LRFN5 and OLFM4, which is worth to be investigated in the future. However, based on our data, it is unclear if the rise in LRFN5 and OLFM4 is a result of MDD or a cause of MDD. Hence, future longitudinal studies assessing serum LRFN5 and OLFM4 levels are necessary to further explore their potential contributions to the pathophysiology of MDD.

There are several limitations to the present study. Firstly, few psychometric data were provided because the study was retrospective in nature. Secondly, we did not observe any other psychiatric comorbidities in the included MDD patients, but we still cannot be 100% sure that all of the included MDD patients had no other psychiatric comorbidities. That is because: (i) MDD patients often have some comorbidities, especially anxiety; Previous studies reported that anxiety symptoms frequently coexist with depression symptoms [70, 71]; (ii) no objective methods to diagnose other psychiatric comorbidities, such as anxiety. Then, there may be some under-diagnosis of other psychiatric comorbidities. Thus, the potential effects of psychiatric comorbidities cannot be totally excluded. Thirdly, we did not compare MDD patients against other psychiatric disorders, such as bipolar disorder; further studies should focus on whether the identified discriminative model can be applied to differentiate MDD patients from patients with other such psychiatric disorders. Fourthly, only the levels of LRFN5 and OLFM4 in serum were detected, thus cerebrospinal fluid investigations are needed to better understand the potential role of these biomarkers in the central nervous system, as they can provide detailed insights into intrathecal processes. However, it needs to be noted that in clinical, cerebrospinal fluid taps are only indicated for neurological but not for psychiatric disorders, and would profound ethical assessments. Fifthly, limited by the number of included samples, future studies with larger samples should be conducted to verify the diagnostic performance of both LRFN5 and OLFM4. Sixthly, the significant differences in LRFN5 and OLFM4 levels between DN-MDD group and DT-MDD group only showed that the antidepressants could cause changes in the expression of proteins in the blood, not that the altered proteins are associated with antidepressants. Thus, whether LRFN5 and OLFM4 were implicated in the mechanism underpinning antidepressant therapeutic action is unclear here and needed further exploration.

In conclusion, our study first evaluated the levels of LRFN5 and OLFM4 in the serum of MDD patients. The significantly higher levels of both molecules in MDD patients compared to HCs and in DN-MDD patients compared to DT-MDD patients suggested that LRFN5 and OLFM4 might be implicated in the pathophysiology of MDD and the mechanisms underlying the therapeutic effects of antidepressants. Meanwhile, we found that the combination of these two molecules could yield excellent diagnostic performance in both training set and testing set. Our findings would be helpful for the future development of objective diagnostic methods for MDD and provide fresh perspectives into exploring the pathophysiology of depression.

## Supplementary information

Supplemental Information
