# Supplementary material for: LRFN5 and OLFM4 as novel potential biomarkers for major depressive disorder: a pilot study
Source: Transl Psychiatry. 2023 Jun 6;13:188. doi: 10.1038/s41398-023-02490-7 (PMC10244395; doi:10.1038/s41398-023-02490-7)
Supplement: Supplementary file 1 — Supplemental Information [file 41398_2023_2490_MOESM1_ESM.docx]

**LRFN5 and** **OLFM4 as novel potential biomarkers for major depressive** **disorder: a pilot study**

***Supplemental Information***

**Supplementary tables and figures**


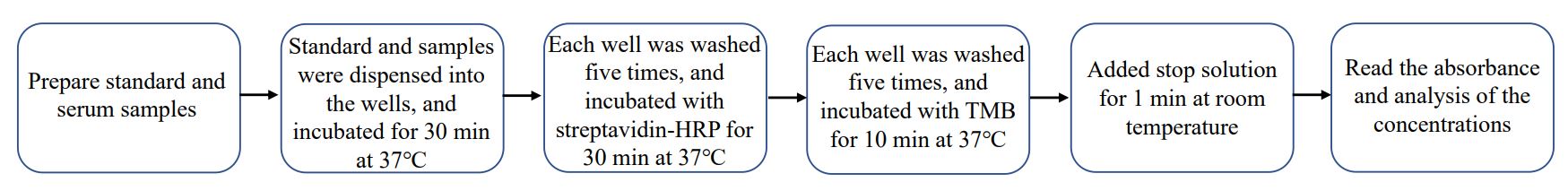


**Fig S1. Flow chart of the ELISA experiment.**

**Table S1 Clinical details of recruited subjects in the study.**

| Characteristics | HCs | MDD | p value |
| --- | --- | --- | --- |
| Sample Size (n)  Sex (Male/Female)  Age (years)  Range  Mean ± SEM  HAMD (Mean ± SEM)  Duration of illness (months)  Range  Mean ± SEM | 81  44/37  23-68  41.988±1.424  4.951±0.289  -  - | 189  91/98  18-69  43.206±1.187  30.418±0.570  0.50-348  46.117±4.359 | -  0.353 ^a^  -  0.768 ^b^  8.67E-39 ^b^  -  - |

Continuous variables are expressed as Mean ± Standard Error of the Mean (SEM).

*HAMD*, hamilton depression scale; *HCs*, healthy controls; *MDD*, major depressive disorder.

^a^ Analyzed by the Chi-square test; ^b^ Analyzed by Mann-Whitney U test.

**Table S2 Characterization of the major depressive patient subjects.**

| Characteristics | DN-MDD (N=99) | DT-MDD (N=90) |
| --- | --- | --- |
| Marital status  Single  Married  Divorced  Widowed  Levels of education  Low  Middle  High  Personal history  Drinking  Never  Moderate  Heavy  Smoking  Never  Moderate  Heavy  Types of antidepressants (n, %)  Fluoxetine  Sertraline  Escitalopram  Olanzapine  Mirtazapine  [Venlafaxine](javascript:;)  Others | 30 (30.30%)  64 (64.65%)  2 (2.02%)  3 (3.03%)  35 (35.35%)  19 (19.19%)  45 (45.45%)  88 (88.89%)  10 (10.10%)  1 (1.01%)  85 (85.86%)  9 (9.09%)  5 (5.05%)  NA  NA  NA  NA  NA  NA  NA | 29 (32.22%)  52 (57.78%)  7 (7.78%)  2 (2.22%)  33 (33.04%)  24 (40.63%)  33 (26.34%)  75 (81.25%)  14 (14.29%)  1 (4.46%)  73 (83.93%)  15 (13.39%)  2 (2.68%)  *Total number =* 122*^#^*  12 (9.84%)  15 (12.30%)  25 (20.49%)  25 (20.49%)  13 (10.66%)  21 (17.21%)  11 (9.02%) |

Highest achieved educational level was determined and defined in three groups for analysis: low level of education (medium-level secondary education or below), medium level of education (higher-level secondary education or vocational education), and high level of education (diploma level or university education).

*DN-MDD*, drug-naïve major depressive disorder; *DT-MDD*, drug-treatment major depressive disorder.

^#^ Some patients used more than one antidepressant.


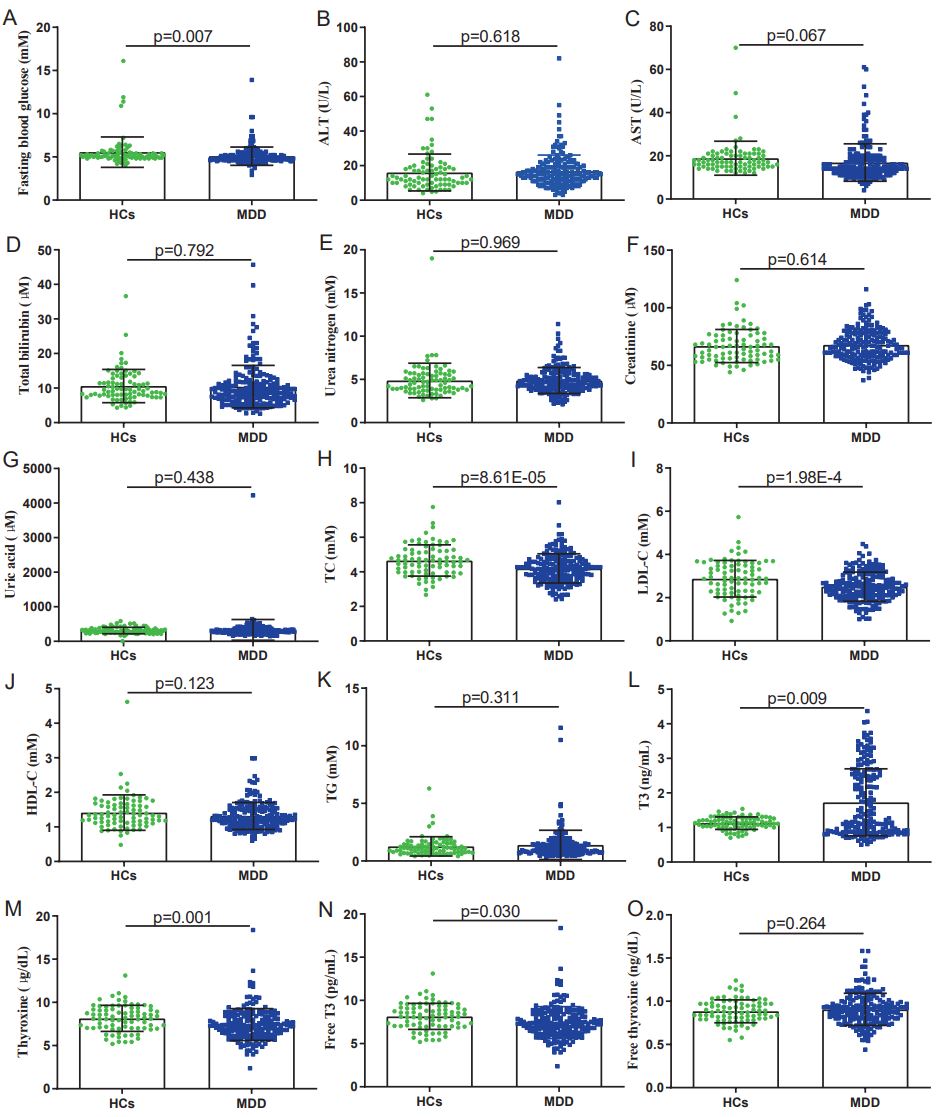


**Fig S2. Concentrations of fasting blood glucose (A), alanine aminotransferase (ALT; B), aspartate aminotransferase (AST; C), total bilirubin (D), urea nitrogen (E), creatinine (F), uric acid (G), total cholesterol (TC; H), low-density lipoprotein cholesterol (LDL-C; I), high-density lipoprotein cholesterol (HDL-C; J), triglyceride (TG; K), triiodothyronine (T3; L), thyroxine (M), free T3 (N), and free thyroxine (O) in healthy controls (HCs) and major depressive disorder (MDD) groups.** Data are presented as mean ± S.D.


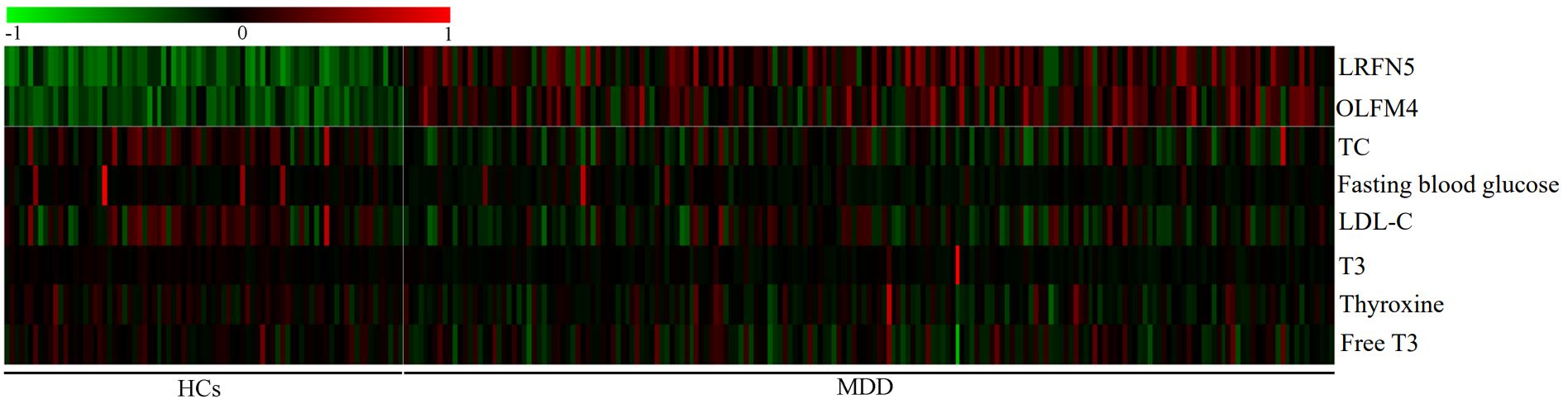


**Fig S3. Differential variables between MDD patients and HCs.** *HCs*, healthy controls; *LDL-C*, low-density lipoprotein cholesterol; *MDD*, major depressive disorder; *TC*, total cholesterol; *T3*, triiodothyronine.

**
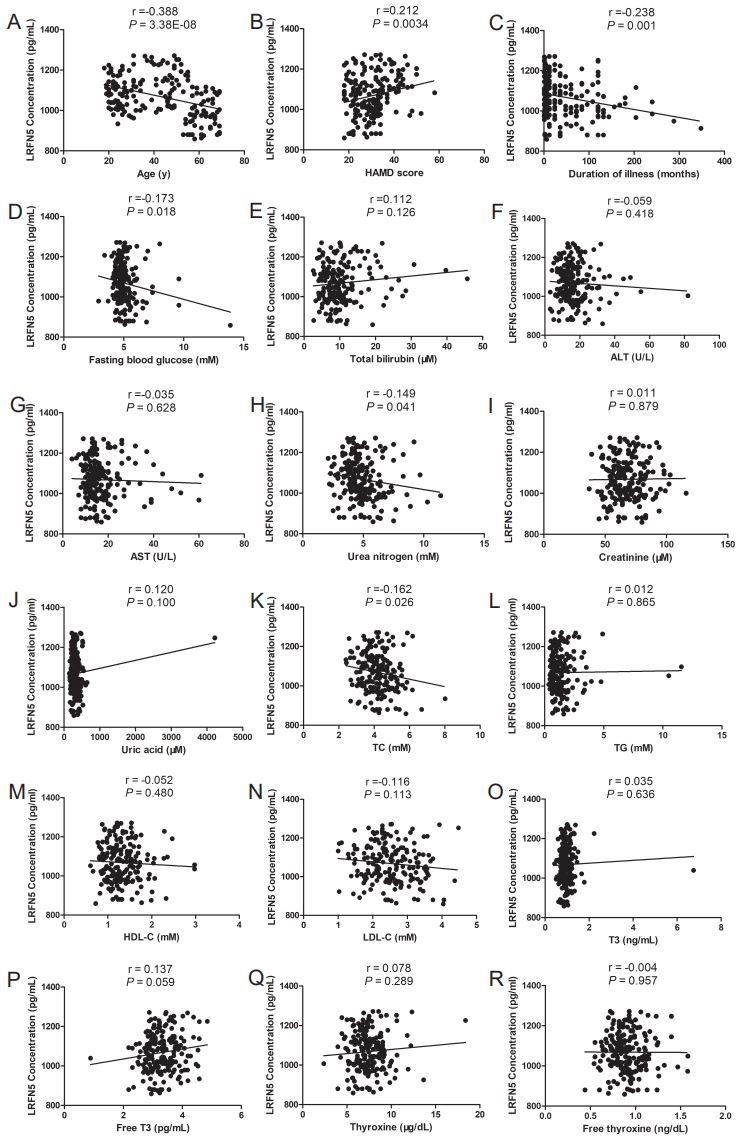
**

**Fig S4. Pearson correlation between age (A),** **Hamilton Depression Scale (****HAMD) score (B), duration of illness (C), fasting blood glucose (D), total bilirubin (E), alanine aminotransferase (ALT; F), aspartate aminotransferase (AST; G), urea nitrogen (H), creatinine (I), uric acid (J), total cholesterol (TC;** **K), triglyceride (TG; L), high-density lipoprotein cholesterol (HDL-C; M), low-density lipoprotein cholesterol (LDL-C; N), triiodothyronine (T3;** **O), free T3 (P), thyroxine (Q), and free thyroxine (R) concentrations and LRFN5 level in major depressive disorder patients.**

**
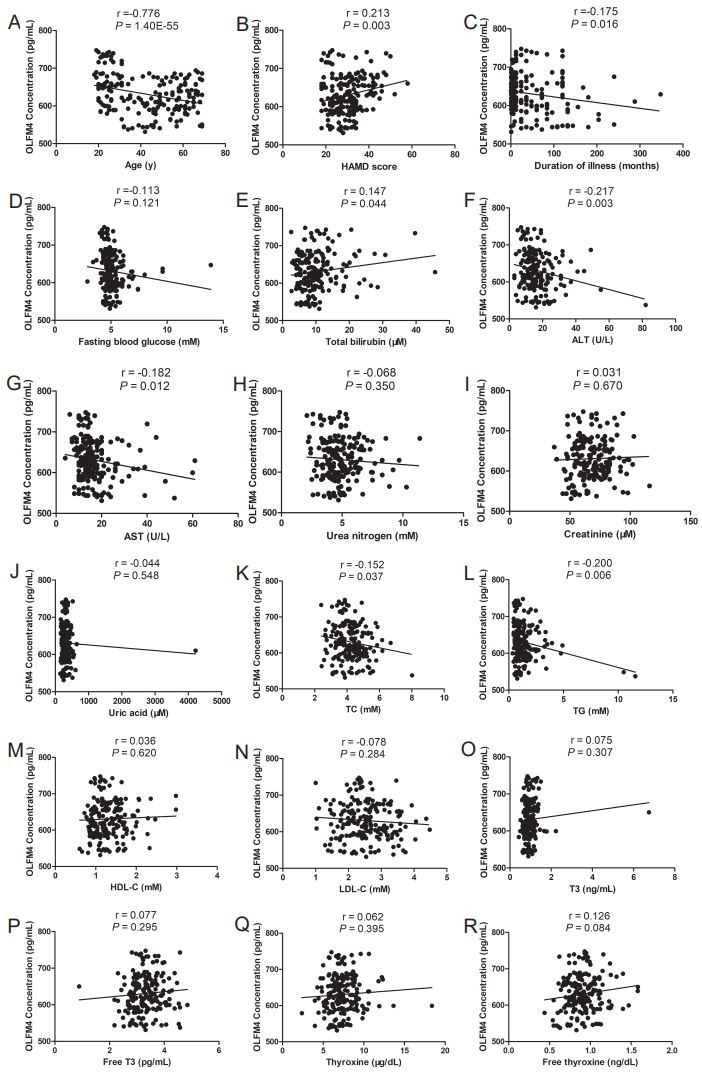
**

**Fig S5. Pearson correlation between age (A), Hamilton Depression Scale (HAMD) score (B), duration of illness (C), fasting blood glucose (D), total bilirubin (E), alanine aminotransferase (ALT; F), aspartate aminotransferase (AST; G), urea nitrogen (H), creatinine (I), uric acid (J), total cholesterol (TC;** **K), triglyceride (TG; L), high-density lipoprotein cholesterol (HDL-C; M), low-density lipoprotein cholesterol (LDL-C; N), triiodothyronine (T3;** **O), free T3 (P), thyroxine (Q), and free thyroxine (R) concentrations and OLFM4 level in major depressive disorder patients.**

**Table S3 Characteristics of the antidepressants used in DT-MDD patients**

| Characteristics | Single | antidepressant |  |  |  |
| --- | --- | --- | --- | --- | --- |
|  | SSRI | Other |  | Combined antidepressants | p value |
| Sample Size (n)  Sex (Male/Female)  Age (years)  Range  Mean ± SEM  HAMD (Mean ± SEM)  Duration of illness (months)  Range  Mean ± SEM | 30  14/16  20-69  39.367±2.689  27.700±0.992  1-204  67.967±11.671 | 21  9/12  20-65  42.571±3.531  27.905±0.993  1-204  55.000±11.563 |  | 39  20/19  21-69  45.103±2.565  26.744±0.724  1-348  68.487±12.858 | -  0.814 ^a^  -  0.249 ^b^  0.486 ^b^  -  0.805 ^b^ |

Continuous variables are expressed as Mean ± Standard Error of the Mean (SEM).

*DT-MDD*, drug-treatment major depressive disorder; *HAMD*, hamilton depression scale; *HCs*, healthy controls.

*SSRI*, selective serotonin reuptake inhibitors.

^a^ Analyzed by the Chi-square test; ^b^ Analyzed by Kruskal-Wallis H test.
